# DOCK8 mutation diagnosed using whole-exome sequencing of the dried blood spot-derived DNA: a case report of an Iraqi girl diagnosed in Japan

**DOI:** 10.1186/s12881-019-0837-4

**Published:** 2019-06-26

**Authors:** Lika’a Fasih Y. Al-Kzayer, Hanadi Munaf H. Al-Aradi, Tomonari Shigemura, Kenji Sano, Miyuki Tanaka, Motoharu Hamada, Kenan Hussien Ali, Osamah Mohammed Aldaghir, Yozo Nakazawa, Yusuke Okuno

**Affiliations:** 1 Department of Pediatrics, Shinshu University School of Medicine, Matsumoto, Nagano, Japan; 2 Department of Pediatrics, Al-Hussein Teaching Hospital, Samawah, Muthanna Iraq; 3 Department of Pathology, Iida Municipal Hospital, Iida, Nagano, Japan; 4 Department of Pediatrics, Nagoya University Graduate School of Medicine, Nagoya, Japan; 5 Department of Family Medicine, Baghdad University, College of Medicine, Baghdad, Iraq; 6 Department of Oral and Maxillofacial Surgery, College of Dentistry, Al-Muthanna University, Samawah, Muthanna Iraq; 7 Center for Advanced Medicine and Clinical Research, Nagoya University Hospital, 65 Tsurumai-cho, Showa-ku, Nagoya, 466-8560 Japan

**Keywords:** DOCK8 deficiency, Primary immune deficiency (PID), Dried blood spots, Flinders technology associates (FTA) cards, Whole exome sequencing (WES), Hyper immunoglobulin E syndrome (HIES), Lymphoproliferative disease (LPD), Consanguineous marriages, Iraq

## Abstract

**Background:**

Dedicator of cytokinesis 8 (DOCK8) deficiency (MIM #243700) is a rare disease, leads to a combined primary immunodeficiency (PID), and accounts for the autosomal recessive-hyper immunoglobulin E syndrome (AR-HIES). *DOCK8* deficiency status characterizes by recurrent infections, atopy, and risk of cancer. Lymphoproliferative disease complicating PID, is difficult to diagnose. Our aim is to present a rare case of PID, and to the best of our knowledge, she is the first case of *DOCK8* deficiency from Iraq. The genetic diagnosis was carried out in Japan using dried blood spot-based DNA transfer and whole-exome sequencing.

**Case presentation:**

An 11-year-old Iraqi girl, of double first-cousin-parents, had a history of severe eczema, food allergy, and repeated infections. She presented with a jaw mass, bilateral cervical and axillary lymphadenopathy, and immunoglobulin (Ig) assays of 20, 3.3 and 1.7-fold above maximum normal level for age of IgE, IgA and IgG, respectively, along with a low IgM, eosinophilia and lymphopenia. Based on the jaw mass biopsy, non-Hodgkin lymphoma was suggested in Iraq, whereas histopathological re-evaluation in Japan revealed the diagnosis of a polyclonal reactive proliferation spectrum of lymphoproliferative disorders/plasmacytic hyperplasia, complicating PID. Whole-exome sequencing supported the diagnosis of PID by identifying a homozygous *DOCK8* mutation with previously reported pathogenicity (NM_203447:c.3332delT, p.Phe1113Leufs*2), that may be attributed to consanguinity.

**Conclusions:**

International collaboration using an effective DNA transportation technique and next-generation sequencing was the key to pinpoint the diagnosis of *DOCK8* deficiency. Our case asserted that careful pathogenetic evaluation, in an advanced setting, was crucial for ruling out the neoplastic process. Pediatricians in areas with a high prevalence of consanguinity marriage should have a high index of suspicion of *DOCK8* deficiency in patients with recalcitrant eczema, and frequent respiratory and skin infectious episodes.

**Electronic supplementary material:**

The online version of this article (10.1186/s12881-019-0837-4) contains supplementary material, which is available to authorized users.

## Background

Hyperimmunoglobulin E syndromes (HIES) are rare diseases among primary immunodeficiency (PID) disorders, characterized by elevated immunoglobulin (Ig) E level, eosinophilia, and recurrent Staphylococcal infections. The autosomal dominant (AD)-HIES is caused by signal transducer and activator of transcription 3 (STAT3) mutations [1, 2]. Bi-allelic loss-of-function mutations in the guanine-nucleotide exchange factor dedicator of cytokinesis 8 (DOCK8) cause autosomal recessive (AR)-HIES [3–5]. *DOCK8*, encoding a protein implicated in the process of regulation of actin cytoskeleton, plays an important role in T/B-cell development and functions, as well as in *STAT3* activation [6–8]. *DOCK8* deficiency (MIM #243700) leads to combined immunodeficiency, rendering the affected patients prone to viral, fungal, and bacterial infections, with various devastating sequelae related to infections, atopy, and malignancy [1, 6, 8]. The possible infectious episodes in DOCK8-deficient status include repeated respiratory infections, and extensive cutaneous viral infections including (Herpes simplex, Herpes zoster, Molluscum Contagiosum, and Human papillomavirus), in addition to *Staphylococcus aureus* skin infections, and mucocutaneous candidiasis [3, 4]. Atopic dermatitis and food allergies are associated with *DOCK8* deficiency [1, 7]. Furthermore, hepatic disorders such as sclerosing cholangitis and hepatitis, are possible associated illnesses [9]. DOCK8-deficient patients are at risk of malignancy such as squamous cell carcinoma and lymphoma [1, 3]. Management of *DOCK8* deficiency comprises screening for, and treatment of complications, as well as administration of antiviral, antifungal, and antibacterial prophylaxis, along with immunoglobulin replacement. However, currently, the only curative therapy is hematopoietic stem cell transplantation (HSCT), which results in immune recovery and reversal of atopic and infectious complications [6, 10, 11].

Whole-exome sequencing (WES) is an advanced approach, covers > 95% of the exons which harbor most of the genetic variants associated with phenotypes of human diseases [12]. Likewise, Flinders Technology Associates (FTA) cards are convenient for dried blood spots (DBS) archiving, transportation, DNA/RNA extraction and further genetic analysis [13–16].

The purpose of this paper is to report a case of *DOCK8* deficiency in an Iraqi girl who had been clinically diagnosed as having HIES, with suspicion of non-Hodgkin lymphoma (NHL), in Iraq. FTA cards were used to transfer her bone marrow aspirate (BMA), and WES was performed, along with re-evaluation of her biopsy specimen, in Japan. To the best of our knowledge, this is the first case report of *DOCK8* deficiency from Iraq.

## Case presentation

Our patient is an 11-year-old girl of double first-cousin parents (first cousins from both maternal and paternal sides), from Muthanna, Southern Iraq. Since the first 2 years of her life, she had a history of food allergy (egg and peanuts), and severe eczematous skin lesion which was resistant to local and systemic steroids. She also had repeated sinopulmonary infections and were often treated in an outpatient setting. Moreover, recurrent infection with molloscum contagiosum and flat warts on the face, neck, behind ears, axillary area and genitalia, were encountered. Notably, she had a history of dental problems related to malocclusion and retention of primary teeth, necessitating dental intervention, in addition to mucocutaneous candidiasis. Vaccinations were given according to schedule in Iraq. At 9-year-old, the patient presented with a slowly growing right jaw mass and toothache, with no history of fever, headache or bone pain. Antibiotics were used, yet the mass continued to increase slowly in size over several months without a change in the overlying skin. Upon examination, she had coarse facies with eczematous scaly itchy skin lesion distributed over her face, scalp, and body as well as genitalia. A non-tender right jaw swelling was evident, with a right submandibular lymph node (2.5 cm), and bilateral cervical and axillary lymphadenopathy (1.5–2 cm). Oral examination showed a fungating mass related to right mandible with a bad odor. Otherwise, no dysmorphic facies, jaundice, fever, café au lait spots, or edema was observed. Scattered crepitation, and a palpable liver were evident by chest and abdominal examination, respectively, whereas, neurological and musculoskeletal examination was normal. Her growth parameters were below 3rd-centile, yet her school performance was good. Although, not genetically determined, her older brother shared with our case similar but milder clinical features of *DOCK8* deficiency. On the other hand, her younger brother was phenotypically diagnosed with Crigler–Najjar syndrome.

**Fig. 1:**
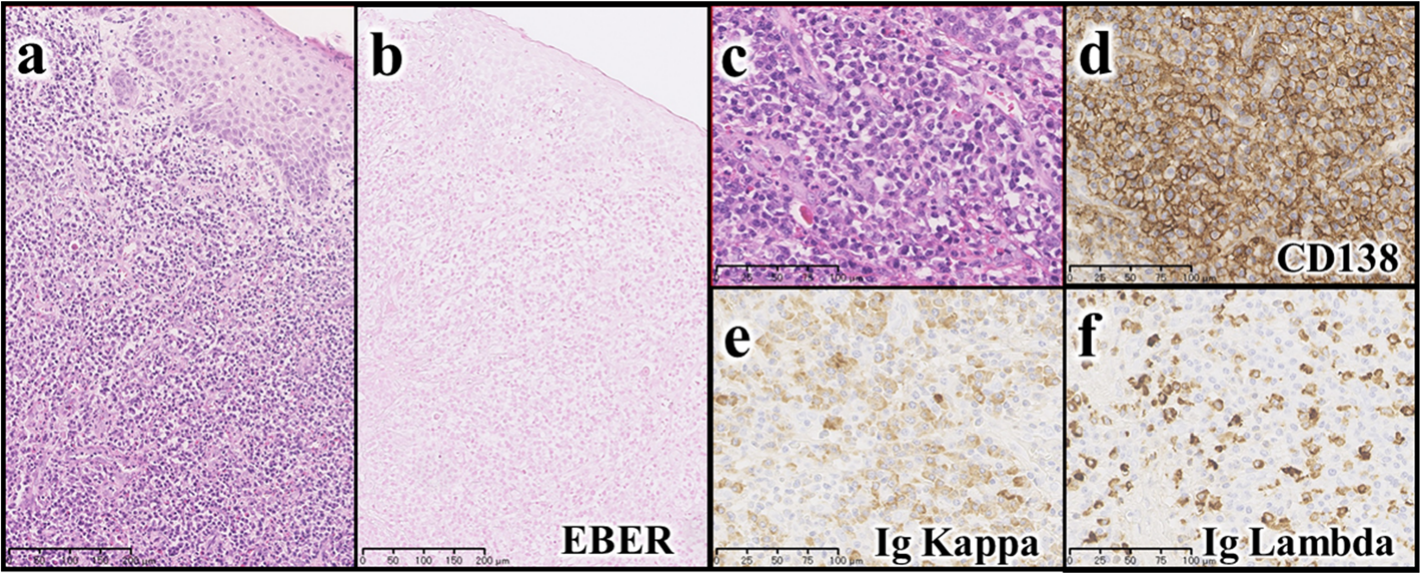
Histopathological evaluation of the jaw mass**. (a)** Hematoxylin and eosin (H&E) staining (low magnification) shows mature plasma cells and lymphocytes densely infiltrated beneath the oral epithelium. **(b)** No EBV infection is detected by EBV-encoded small RNA (EBER)-in situ hybridization. **(c)** H&E staining (high magnification) shows remarkable mature plasma cell infiltration. **(d)** Most of the cells are CD138-positive phenotype (indicating plasma cells). **(e)** Positive Ig kappa. **(f)** Positive Ig lambda chain expression

During the assessment for the jaw swelling, laboratory investigations in Iraq showed a marked eosinophilia and lymphopenia, along with Ig assays of 20, 3.3 and 1.7-fold above maximum normal level for age of IgE, IgA and IgG, respectively, whereas IgM was low (Table 1). She also had hypercellular BMA with eosinophilia, iron deficiency anemia, reactive thrombocytosis, and elevated erythrocyte sedimentation rate and lactate dehydrogenase levels, as well as deranged liver function, and a high anti-tissue transglutaminase (anti-tTG) of 10-fold above normal. Meanwhile, two-biopsies were taken from the jaw mass, and NHL was suggested in Iraq. One of the biopsy specimens was re-evaluated in Japan. The immunostaining in Japan revealed that most of the tissue was plasma cells with positive-CD138, along with Ig-kappa and lambda chain expressions, and few small lymphocytes showing the polyclonal pattern (Fig. 1). Epstein-Barr virus (EBV) was undetectable by in situ hybridization (ISH) in Japan. Furthermore, no other significant etiological microorganisms including human herpes virus 8, bacteria, or fungi were detected in the tissue after using immunohistochemistry, Gram staining, Grocott staining, and acid-fast staining. Based on the finding that neither light chain deviation in plasma cells was apparent (Figs.1e and f), nor PCR clonality in Ig-heavy chain complementarity determining region (CDR)-III was disclosed, the lymphoplasmacytic proliferation in the mass was interpreted as non-neoplastic/reactive process. Thus, the diagnosis of a polyclonal reactive proliferation spectrum of lymphoproliferative disease (LPD) complicating PID was made in Japan. Concurrently, FTA cards were used to transfer a few drops of the patient’s BMA to Japan, where the DNA was extracted from the DBS on FTA filter-paper and was subjected to WES [16]. A *DOCK8* homozygous frameshift deletion (NM_203447:c.3332delT, p.Phe1113Leufs*2) was detected in a region of run of homozygosity in chromosome 9p, suggesting that the mutation became homozygous because of consanguinity [17].

**Table 1:** Laboratory data of the case with DOCK8 deficiency from Iraq

Variable (unit)	Patients’ value	Normal value
Complete Blood Count		
White blood cells (WBC)		
Total WBC (× 10^9^/L)	12.5	5–14.5
Differential WBC (%)		
Neutrophil	43.5	33–76
Lymphocyte	29.8	35–61
Monocyte	5.8	0–5
Eosinophil	19.6	0–3
Basophil	1.3	0–1
Hb (g/dl)	10.7	12–15
MCV (fL)	70.2	77–95
MCH (pg)	20.4	25–33
MCHC (g/dl)	29.1	32–36
Platelets (× 10^9^/L)	575	150–450
Immunoglobulin Assay		
IgE (I.U./ml)	> 4000	< 200
IgG (mg/dl)	2587	600–1500
IgA (mg/dl)	755	35–230
IgM (mg/dl)	18	40–180

Therefore, the patient was diagnosed as *DOCK8* deficiency, and the jaw swelling was managed conservatively, through applying strict oral hygiene measures as recommended by the maxillofacial surgeon, who also performed frequent superficial debridement to remove any necrotic tissue, along with extraction of any deciduous tooth that was badly carious. Moreover, antimicrobial agents (amoxiclav and co-trimoxazole, in addition to metronidazole and nystatin oral drops) were used. Concomitantly, a gluten-free diet was firmly introduced for the management of autoimmune enteropathy (Celiac disease) that was diagnosed serologically in accordance with World Gastroenterology Organization guidelines in countries with limited resources [18].

**Fig. 2:**
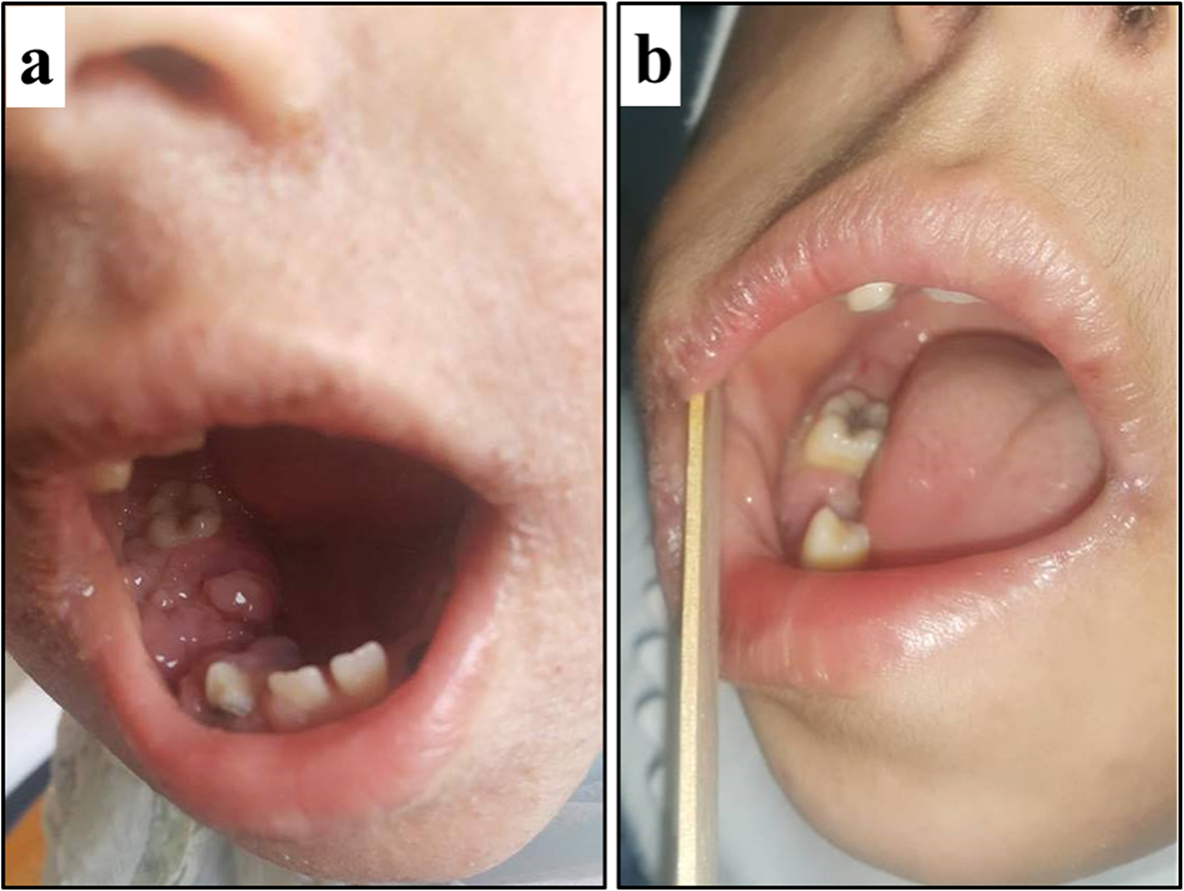
Regression of the jaw mass. **(a)** A right jaw mass is shown from inside the mouth at the age of 9 years. The eczema is evident in the face. **(b)** After 1.5 years of conservative treatments including a gluten-free diet, the mass regressed

Eventually, her jaw mass regressed gradually over several months using neither steroid nor chemotherapy (Fig. 2). Considering the difficult socioeconomic status of the family and the unhealthy condition of the 2 brothers, the option of HSCT was excluded (Additional file 1).

## Discussion and conclusions

Our DOCK8-deficient patient is the second child among three ill siblings as a consequence of a double first-cousin consanguineous marriage. Many of the reported DOCK8-deficient cases were among consanguineous populations, including those of Arabic and Turkish descent [6]. Notably, consanguineous marriages in Middle East countries such as Saudi Arabia, Iraq, Kuwait, Syria and Iran, are exceeding 50% of marriages [16, 19, 20]. *DOCK8* deficiency accounted for a significant proportion (15%) of patients who suffered from combined PID in Kuwait [20].

The unfortunate circumstances of repeated wars and unstable security in Iraq have adversely affected the health sector, leading to a deterioration in the diagnostic/therapeutic services. Based on clinical evaluation, our patient was initially diagnosed in Iraq as a case of Job syndrome (original name of AD-HIES). Given that the clinical features of AD-HIES and AR-HIES are overlapping, it is often difficult to assign patients to one category or another without genetic assessment. Although recurrent sinopulmonary and cutaneous viral infections, as well as food allergy, are characteristic of *DOCK8* deficiency, our patient demonstrated some overlapping features of *STAT3* mutation, such as retained primary teeth [1, 6, 7, 10]. Since our case was diagnosed with HIES, NHL was possible, as suggested by the pathologist in Iraq, however, re-evaluation in Japan had ruled-out malignancy.

It was reported that some disorders present with generalized lymphadenopathy that initially suggest a malignant process but later found to be benign. Many of these uncommon lymphoproliferations are related to an abnormal immune response to some inciting stimuli [21]. Indeed, in the setting of PID, truly malignant neoplasms are sometimes difficult to be differentiated from nonmalignant LPD with aggressive features.

B-cell plasmacytic hyperplasia as a type of lymphoproliferation-associated with PID, was categorized by WHO according to the paradigm of posttransplant LPDs. However, a recently published report pointed out that WHO terminology had skipped and poorly defined other emerged novel types of immunodeficiency-associated-LPDs (IA-LPDs). Likewise, EBV-negative B-cell, T and NK-cell LPDs associated with PID, are rare and less well characterized [22, 23].

Recently, a lymphoproliferation entity related to PID was described and correlated with autoimmunity process, attributed to *STAT3*-gain-of-function mutation; however, the latter works in a different direction compared to *STAT3*-loss-of-function mutation responsible for HIES. Of note, STAT3, which plays an important role in immune regulation, is mediated in terms of activation and signaling by its interaction with DOCK8 protein [24–26].

According to literature, most cases of immunodeficiency-associated B-cell hyperplasia, including those of EBV-positive type, regress spontaneously. Surgical excision can be helpful for obstructive oral masses [22].

We believe that an intraoral indigenous bacterium was the possible etiological candidate for the lesion in our patient, additionally, the hyperplastic features of the overlying epithelium and the scattered ulceration suggested that the lesion was chronically and locally stimulated. Virtually, chronic gingival trauma and exaggerated inflammatory reaction could be the consequences of HIES-associated dental problems and poor oral hygiene. After excluding the neoplastic or specific infectious etiology in our patient, the comprehensive dental care along with the improvement of oral hygiene mostly contributed to the spontaneous regression of the jaw mass.

The autoimmune enteropathy in our case could be part of the autoimmune manifestations that were previously described in DOCK8-deficient patients [6, 8]. After introducing gluten-free diet, the girl was clinically improving in terms of skin lesion, which was mostly attributed to the overwhelming dermatitis herpetiformis from Celiac disease. Furthermore, her growth parameters had improved, from measuring below 3rd-centile to be on the 5th-centile, along with normalization of the anti-tTG. Meanwhile, the disclosure of *DOCK8* mutation was a diagnostic entity, but unfortunately, HSCT was impossible due to the absence of a suitable donor.

The use of WES of the DBS-derived-DNA was the best approach to reach the diagnosis in our case. We previously utilized FTA cards to transfer samples of Iraqi children with acute leukemia and other hematological diseases [13–16].

In conclusion, international collaboration using FTA cards and next-generation sequencing was helpful to diagnose *DOCK8* deficiency. Moreover, our case asserted that careful pathogenetic evaluation, in an advanced setting, was crucial for ruling out the neoplastic process. Pediatricians in areas with a high prevalence of consanguinity marriage should have a high index of suspicion of *DOCK8* deficiency in patients with recalcitrant eczema, and frequent respiratory and skin infectious episodes.

## Additional file

Additional file 1: Time-line table: Summary of the child’s clinical course with the family pedigree. (DOCX 92 kb)

## Data Availability

The datasets used and analyzed in this study are available from the corresponding author upon reasonable request.

## Abbreviations

AD: Autosomal dominant
Anti-tTG: Anti-tissue transglutaminase
AR: Autosomal recessive
BMA: Bone marrow aspirate
DBS: Dried blood spots
DOCK8: Dedicator of cytokinesis 8
EBER: Epstein-Barr virus-encoded small RNA
EBV: Epstein-Barr virus
FTA: Flinders Technology Associates
HIES: Hyperimmunoglobulin E syndromes
HSCT: Hematopoietic stem cell transplantation
IA-LPDs: Immunodeficiency-associated lymphoproliferative disorders
ISH: In situ hybridization
LPD: Lymphoproliferative disease
PCR: Polymerase chain reaction
PID: Primary immunodeficiency
STAT3: Signal transducer and activator of transcription 3
WES: Whole exome sequencing
