# Supplementary material for: DOCK8 mutation diagnosed using whole-exome sequencing of the dried blood spot-derived DNA: a case report of an Iraqi girl diagnosed in Japan
Source: BMC Med Genet. 2019 Jun 26;20:114. doi: 10.1186/s12881-019-0837-4 (PMC6595679; doi:10.1186/s12881-019-0837-4)
Supplement: Supplementary file 1 — Time-line table: Summary of the child’s clinical course with the family pedigree. (DOCX 92 kb) [file 12881_2019_837_MOESM1_ESM.docx]

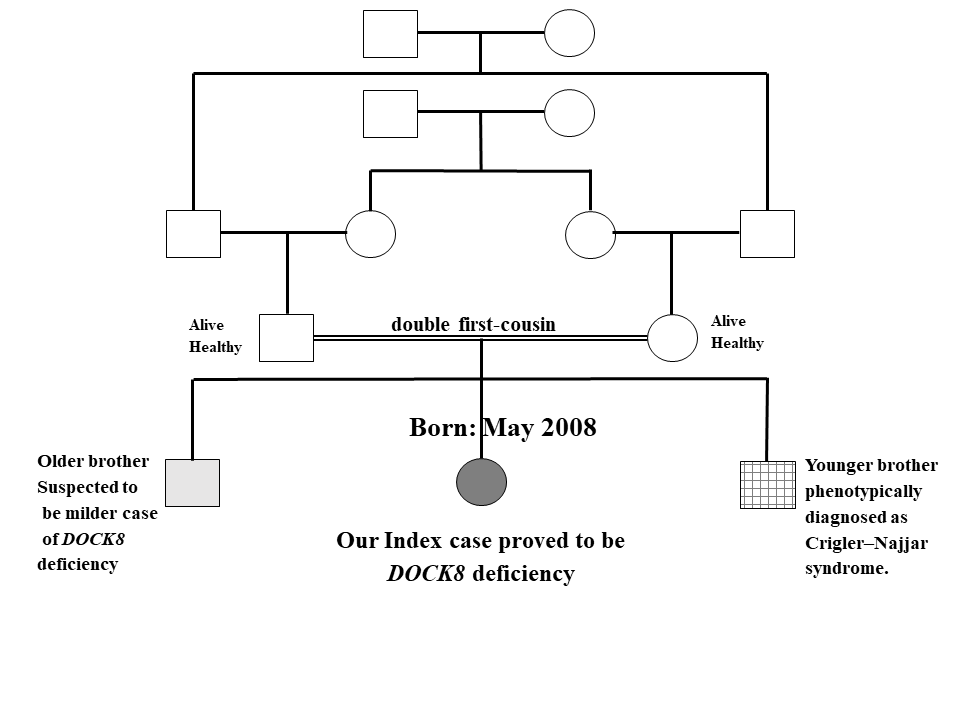


| **Age** | **Date** | **Clinical information** | **Management** |
| --- | --- | --- | --- |
| **1.8 year** | **March 2010** | Severe eczema and repeated respiratory and skin infections  One time she had pneumonia. | Sinopulmonary infections: an average of 4 times a year and were often treated in an out-patient setting with antibiotics. |
| **2 years** | **May 2010** | Food allergy to egg and peanuts. | Avoid egg and peanuts. |
| **5 years** | **2013** | Skin lesion and eczema became sever. | Steroid was used for eczema. |
|  |  | Viral skin infections started and were recurrent. | Diagnosed as Job Syndrome (AR-HIES).  Treated by a dermatologist using topical therapy. |
| **9 years** | **March 2017** | Slowly growing right jaw mass and toothache, with bilateral cervical and axillary lymphadenopathy.  Celiac disease was suspected because of a positive family history of Celiac disease, confirmed iron deficiency anemia, and patients’ growth parameters < 3rd centile, along with the overwhelming itchy skin lesion resembling dermatitis herpetiformis. | Antibiotics were used initially with no response.  Two biopsies were taken from the jaw mass, and the pathologist in Iraq suggested non-Hodgkin lymphoma (NHL). |
| **9.5 year** | **Dec 2017** | The jaw mass continued to increase slowly in size over several months period.  Lab tests: CBC showed marked eosinophilia and lymphopenia, as well as hypochromic microcytic anemia and increased platelets.  Immunoglobulin assays of 20, 3.3 and 1.7-fold above the maximum normal level for age of IgE, IgA and IgG, respectively. Low IgM.  Bone marrow aspirate was hypercellular with eosinophilia.  Low ferritin level.  LDH and ESR were high, with a normal CRP  Liver enzymes were elevated.  Anti-tTG of 10-fold higher than normal | One of the two biopsy specimens was transferred to Japan for re-evaluation. The diagnosis of a polyclonal reactive proliferation spectrum of lymphoproliferative disease (LPD) complicating primary immunodeficiency disorders (PIDs) was made in Japan. Meanwhile, bone marrow sample was transferred as well from Iraq by FTA cards to Japan in form of dried blood spots. Whole-exome sequencing (WES) was done and revealed DOCK8 deficiency.  A gluten-free diet was established. |
| **10.3 year** | **Sep 2018** | The mass regressed gradually over 1.5-year period. | Maxillofacial surgeon recommended strict oral hygiene instructions, including tooth brushing, chlorhexidine mouth wash, and normal saline irrigation. Frequent superficial debridement was done. Antimicrobial agents (antibiotics such as amoxiclav and co-trimoxazole, in addition to metronidazole and nystatin oral drops) were used, as well as the extraction of any deciduous tooth in the area of pathology.  No steroid or chemotherapy was used. |
| **IN JAPAN: 1- DOCK8 deficiency was disclosed by WES.**  **2- Exclusion of NHL or malignancy.**  **3- The jaw mass was diagnosed as a polyclonal reactive proliferation spectrum of**  **LPDs/plasmacytic hyperplasia, complicating PID.** | | | The only curative option, the hematopoietic stem cell transplantation (HSCT), is not possible as both of her brothers are ill. The older brother apparently shared DOCK8 deficiency features, and the younger brother was phenotypically diagnosed as Crigler–Najjar syndrome. |
